# In vivo transplantation of fetal human gut‐derived enteric neural crest cells

**DOI:** 10.1111/nmo.12900

**Published:** 2016-07-06

**Authors:** J. E. Cooper, D. Natarajan, C. J. McCann, S. Choudhury, H. Godwin, A. J. Burns, N. Thapar

**Affiliations:** ^1^ Stem Cells and Regenerative MedicineUCL Institute of Child HealthLondonUK; ^2^ Department of GastroenterologyGreat Ormond Street Hospital NHS Foundation TrustLondonUK; ^3^ Department of Clinical GeneticsErasmus MCRotterdamThe Netherlands

**Keywords:** cell transplantation, enteric nervous system, enteric neuropathies, human enteric neural stem cells, neural crest cells

## Abstract

The prospect of using neural cell replacement for the treatment of severe enteric neuropathies has seen significant progress in the last decade. The ability to harvest and transplant enteric neural crest cells (ENCCs) that functionally integrate within recipient intestine has recently been confirmed by in vivo murine studies. Although similar cells can be harvested from human fetal and postnatal gut, no studies have as yet verified their functional viability upon in vivo transplantation. We sought to determine whether ENCCs harvested from human fetal bowel are capable of engraftment and functional integration within recipient intestine following in vivo transplantation into postnatal murine colon. Enteric neural crest cells selected and harvested from fetal human gut using the neurotrophin receptor p75^NTR^ were lentivirally labeled with either GFP or calcium‐sensitive GCaMP and transplanted into the hindgut of *Rag2* ^−^ */γc* ^−^ */C5* ^−^‐immunodeficient mice at postnatal day 21. Transplanted intestines were assessed immunohistochemically for engraftment and differentiation of donor cells. Functional viability and integration with host neuromusculature was assessed using calcium imaging. Transplanted human fetal gut‐derived ENCC showed engraftment within the recipient postnatal colon in 8/15 mice (53.3%). At 4 weeks posttransplantation, donor cells had spread from the site of transplantation and extended projections over distances of 1.2 ± 0.6 mm (*n* = 5), and differentiated into enteric nervous system (ENS) appropriate neurons and glia. These cells formed branching networks located with the myenteric plexus. Calcium transients (change in intensity F/F0 = 1.25 ± 0.03; 15 cells) were recorded in transplanted cells upon stimulation of the recipient endogenous ENS demonstrating their viability and establishment of functional connections.

**Key Points**

- ENCCs have been identified as a source for a cell replacement therapy in enteric neuropathies. Murine ENCCs are capable of colonization and function within mouse bowel in vivo. This work investigated the potential of human gut‐derived ENCC in vivo.
- Upon transplantation, fetal human‐derived ENCCs display engraftment, spread, extension of projections, differentiation towards neurons and glia and functional connectivity with the endogenous ENS.
- This is the first report of in vivo transplantation of human gut‐derived ENCC and provides proof‐of‐concept data for their clinical application in cell replacement therapies.

## 1 Introduction

Enteric neuropathies are a diverse and clinically important range of conditions characterized by aberrant or absent propulsive contractile activity secondary to loss or malfunction of the enteric nervous system (ENS).^1^, ^2^, ^3^, ^4^ Such disorders range from those in which there is a congenital or acquired absence of intrinsic ENS (e.g. Hirschsprung disease and esophageal achalasia), to conditions such as intestinal pseudo‐obstruction and slow transit constipation which result from more subtle neuronal deficits that remain to be better defined.^5^, ^6^, ^7^ Current treatments are unsatisfactory and limited to surgical interventions, which are associated with high levels of morbidity and mortality.^1^ Alternative therapeutic strategies are required for these conditions.^8^

The ENS is formed during embryogenesis by migratory neural crest cells arising in the vagal and sacral regions of the neural tube (reviewed in Ref. 9). Enteric neural crest cells (ENCC), which give rise to enteric neurons, glia, and stem cells, can be isolated from both embryonic and postnatal murine intestine.^10^, ^11^ Furthermore, upon transplantation, they are able to colonize both ganglionic and aganglionic murine bowel in vivo, forming neural networks that display functional integration with the host neuromusculature.^12^, ^13^

The potential to develop a clinically applicable cell replacement therapy for enteric neuropathies has been validated by the isolation of ENCC from fetal and postnatal human gut^8^, ^14^, ^15^, ^16^ To date, human gut‐derived ENCCs have been transplanted within in vitro models of aganglionosis, showing physical integration and differentiation.^14^, ^15^, ^17^ However, their functional integration remains to be demonstrated within a postnatal in vivo model. Here, we report the efficient isolation of ENCC from human fetal gut and successful engraftment and functional integration following in vivo transplantation into immunodeficient murine gut.

## 2 Materials and Methods

### 2.1 Animals

Animals were maintained, and experiments were performed, in accordance with local approvals and the UK Animals (Scientific Procedures) Act 1986 under license from the Home Office (PPL70/7500). *Rag2* ^−^ */γc* ^−^ */C5* ^−^‐immunodeficient mice, deficient in innate immunity and lacking all lymphocytes, were used as recipients for ENCC transplantations.^18^, ^19^

### 2.2 Human cells and tissues

Fetal human gut (age 12–15 weeks) was obtained from the Human Developmental Biology Resource, UCL Institute of Child Health, London, UK, with informed, written consent and under ethical approval from the Health Research Authority (08/H0712/34+5). Studies were performed according to the Declaration of Helsinki. Enteric neural crest cells were isolated, sorted, and cultured as described previously.^14^ They generated primary neurospheres after approximately 1 week, and these were transplanted within 15–30 days.

### 2.3 Lentiviral labeling of human ENCC

Lentiviral constructs expressing enhanced green fluorescent protein (EGFP)^20^ and GCaMP (Addgene plasmid 42168:pJMK019, Adam Cohen; Addgene, Cambridge, MA, USA) were used to label cells with high levels of GFP or a calcium‐sensitive GFP construct, respectively. Isolation of lentiviral particles and subsequent transduction of cells was conducted according to a protocol described previously.^20^

### 2.4 Transplantation of ENCC into gut in vivo

Enteric neural crest cells were transplanted into the distal colon of immune‐deficient *Rag2* ^−^ */γc* ^−^ */C5* ^−^ knockout mice via laparotomy (described previously Ref. 13) at weaning (postnatal day 21; *n* = 15). Mice were maintained for 4 weeks posttransplantation before they were killed and analyzed.

### 2.5 Immunohistochemistry

Transplanted bowel was fixed and analyzed using immunohistochemistry as described previously.^13^ The primary antibodies used were mouse TuJ1 (1:1000; Biolegend, London, UK) and rabbit anti‐cow S100 (1:500; Dako, Ely, Cambridgeshire, UK), and secondary antibodies were anti‐mouse and anti‐rabbit Alexafluor 568 (1:500; Invitrogen, Carlsbad, CA, USA).

Images were acquired on a Zeiss LSM 710 confocal microscope (Zeiss, Cambridge, UK) and processed using ImageJ^21^ and Adobe Photoshop CS3 software (Adobe, San Jose, CA, USA).

### 2.6 Calcium imaging of transplanted ENCC

Colonic gut samples were prepared as described previously.^13^ The endogenous ENS was stimulated via an electrode placed approximately 200 μm from the transplanted cells. Changes in the fluorescence intensity of GCaMP elicited by calcium transients within transplanted cells were then imaged and processed as described previously.^13^

## 3 Results

**Figure 1:**
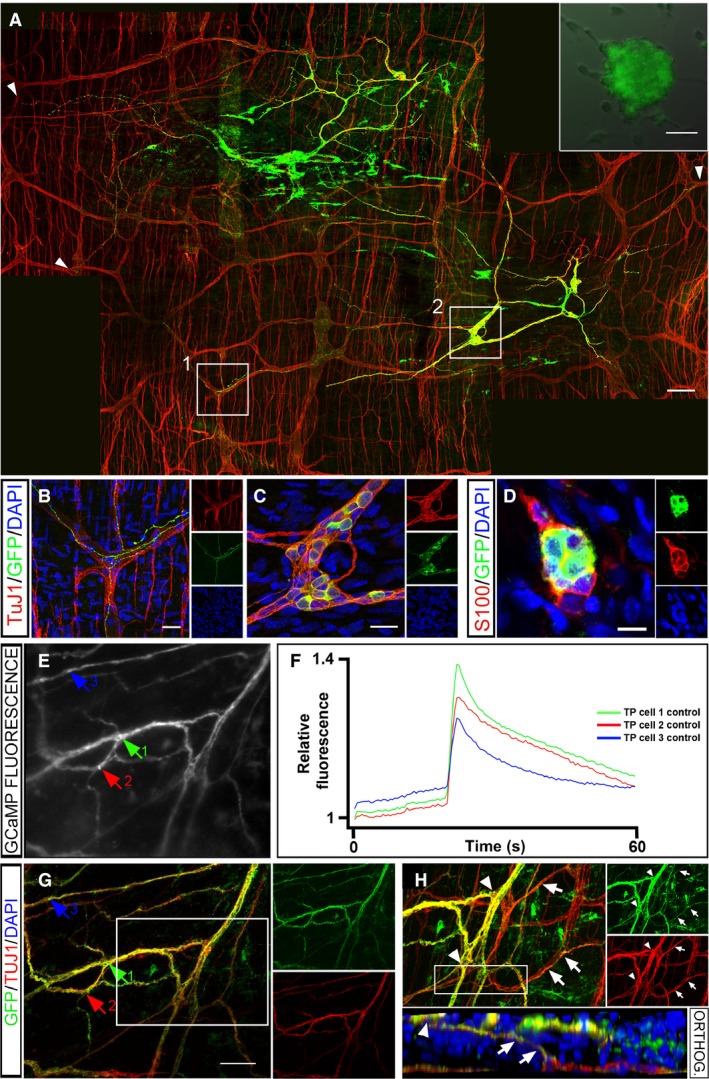
Transplanted GFP/GCaMP5G+ human fetal p75 sorted gut‐derived ENCCs generate functional networks of enteric neurons and glia in mouse gut. (Inset in A) p75+ FACS selected human fetal (week 14) gut‐derived neurosphere transduced with GFP (green). (A) Wholemount gut preparation in which GFP+ transplanted cells (green) project along endogenous TuJ1+ ENS nerve fibers (red). Arrowheads indicate the extent of neuronal projections within the myenteric plexus. (B) High‐power image of TuJ1+ enteric ganglia (red) showing co‐localization with GFP+ cell projections (taken from inset 1 in A). (C) High‐power image of TuJ1+; GFP+ transplanted cells within a ganglion‐like structure containing TuJ1+ cells (taken from inset 2 in A). (D) GFP+ transplanted cells (green) co‐express the glial marker S100 (red). (E) Representative image of transplanted GCaMP5G+ fetal human‐derived neurons within *Rag2* ^−^ */γc* ^−^ */C5* ^−^ mouse distal colon. Arrows indicate neurons from which Ca2^+^ responses are plotted in (F). (F) Representative traces showing Ca^2+^ responses recorded as F/F0 from transplanted neurons (TP cell) upon stimulation of endogenous neuronal fibers. (G) Transplanted cells and fibers indicated by arrows (equivalent to those in E) co‐express GFP and the neuronal marker TuJ1. (H) Z‐projection of the boxed area in G showing GFP/TuJ1 expressing (yellow) transplanted cell bodies (arrowheads) and their projections (arrows) within the endogenous TuJ1 expressing myenteric plexus (red). Inset below shows an orthogonal view (orthog.) taken through the entire depth of the boxed region showing transplanted neuronal cell bodies (arrowhead) projecting (arrowheads) to the endogenous myenteric plexus (red). DAPI labels nuclei in blue. Scale bar in A inset = 50 μm; A = 100 μm; B and C = 25 μm; D = 10 μm; D = 100 μm. Insets in B–D, G, and H show individual channels.

Fetal human gut tissue was dissociated and ENCC selected by FACS, based on the expression of the NCC marker p75 (25.6 ± 2.5% of dissociated cells were p75+ve; *n* = 3). Enteric neural crest cells were transduced with GFP or GCaMP with a labeling efficiency of 92 ± 3% (*n* = 5) and 75 ± 14% (*n* = 5), respectively. Transduced cells maintained fluorescence and formed GFP/GCaMP‐expressing neurospheres after 7–10 days in vitro (inset in Fig. 1A).

Human fetal GFP+ or GCaMP+ labeled cells were transplanted, as neurospheres, into the distal gut of *Rag2* ^−^ */γc* ^−^ */C5* ^−^‐immunodeficient mice and were detected up to 8 weeks later in 8/15 guts examined (53.3%). GFP expression was strong and facilitated visualization of cell bodies and projections posttransplantation. Transplanted ENCCs spread and formed both interconnections between cells (Fig. 1A) and ganglia‐like structures (Fig. 1C) reminiscent of the endogenous ENS.

Four weeks after transplantation, GFP+ cells expressed neuronal and glial markers (TuJ1, Fig. 1A–C and S100, Fig. 1D, respectively). Cell bodies from transplanted cells were located adjacent to those of endogenous neurons (Fig. 1C) and projections from transplanted cells followed endogenous ENS fiber tracts of the myenteric plexus, showing physical integration within the endogenous network. Projections were visible over a distance of 1.2 ± 0.6 mm (*n* = 5), after 4 weeks (Fig. 1A and B).

GCaMP expression in transplanted cells enabled assessment of functional integration within the gut. Spontaneous calcium transients (global and vesicular) were observed in ENCC both in vitro and occasionally in transplanted cells within gut preparations ex vivo (data not shown). Electrical point stimulation applied to endogenous neuronal fibers within the host gut musculature resulted in calcium transients in cell bodies and fibers of transplanted GCaMP+ cells (change in intensity F/F0 = 1.25 ± 0.03; 15 cells) (Fig. 1E and F and Video S1). Subsequent TuJ1 immunohistochemical staining confirmed that GCaMP+ cell bodies and fibers, activated upon stimulation of the endogenous network, were neuronal (Fig. 1G). 3D reconstruction of confocal images revealed the fine structure of the physical connections of the transplanted human‐derived GCaMP+ neurons and the endogenous ENS (Fig. 1H).

## 4 Discussion

A number of studies using murine cells support the idea of using stem cell transplantation for the treatment of enteric neuropathies such as Hirschsprung disease.^12^, ^13^, ^14^ As a first step to explore the potential of human gut‐derived ENCC for in vivo transplantation, we sought to study fetal enteric neural stem cells. We have previously shown that neurospheres containing such cells are formed faster and with higher efficiency than their postnatal counterparts.^16^ We wished to confirm that committed human ENCCs sourced early in development retain the potential for cell therapy. To this end, we demonstrated that fetal gut‐derived ENCC can be efficiently isolated from the enteric cell population by sorting for p75 (25.6 ± 2.5%), with a greater efficiency than that achieved by ourselves and others for postnatal gut‐derived ENCC (5.8 ± 2.8%; D. Natarajan, unpublished findings and 4.82; SEM = 1.43%, respectively).^22^ These ENCC can be labeled using lentiviral constructs to facilitate subsequent cell tracing and functional studies. Human fetal ENCC proved successful at colonizing postnatal mouse colon, forming extensive networks of neurons and glia, which physically integrated with the endogenous ENS. Crucially we showed, for the first time, the functional integration of transplanted human gut‐derived cells within in vivo gut.

Having confirmed the ability of fetal human gut‐derived ENCC to functionally colonize the intact ENS, a number of challenges remain to be addressed. These include determining whether these cells retain functionality and elicit rescue within a model of enteric neuropathic disease, if they are safe to transplant and indeed whether they are a viable cell source for use in a therapeutic setting. Previously, we reported higher cell engraftment outcomes following in vivo transplantation of mouse‐derived ENCC (90.3%) in contrast to 53.3% in this study. This is likely to reflect both our experience that human cells appear somewhat less robust under experimental conditions than their mouse counterparts as well as the expected inherent variability of human samples utilized in our study (e.g., the age and condition of the sample received, gut regions available for culture, and genetic variability between samples). There was no evidence of host immune response in the examined gut (data not shown) to explain the reduced engraftment of human‐derived cells.

A recent study by Fattahi et al. reported the transplantation of human embryonic stem cells (ESC), differentiated toward an early vagal neural crest lineage, in the postnatal colon of a mouse model of Hirschsprung Disease in vivo.^23^ Although the transplanted cells appeared to show efficient pan‐colonic colonization and were able to rescue the lethal phenotype of this model, the authors were unable to comment on the mechanism by which rescue may have been elicited and functionality of the transplanted cells was not demonstrated.^23^ Although their work is a step toward validation of the use of ESC in enteric neuropathies, ESC safety for transplantation, in terms of colonizing off‐target locations and tumorigenicity, was not addressed. In our study, we successfully harvested a cell type already committed to the ENS lineage, theoretically reducing the risk of uncontrolled proliferation. This is supported by our previous work using mouse‐derived ENCC, which provided evidence that this cell type is safe to transplant within an in vivo environment.^13^ Such safety data must be further extended to human gut‐derived ENCC.

Here, we used fetal human gut‐derived ENCC as a cell source with which to demonstrate the ability of human ENCC to integrate functionally with the ENS in vivo. Given concerns regarding the sourcing and use of fetal (and embryonic) cells as a therapeutic tool, our current studies aim to investigate the capabilities of postnatally sourced ENCC within the in vivo environment. We and others have demonstrated that ENCC can be sourced from routine mucosal gut biopsies of both children and adults.^14^, ^24^, ^25^ Clinically, these cells could benefit from being autologously sourced from minimally invasive procedures, thus circumventing both ethical concerns and any requirement for immunosuppression upon transplantation. However, several challenges remain to be addressed including optimization of protocols to maximize the proliferative capacity of postnatal‐derived cells, and convincing demonstration of their functional viability.

In conclusion, our findings advance the search for a cell replacement therapy for enteric neuropathies by demonstrating that within the context of in vivo transplantation human gut‐derived ENCCs are able to contribute to the ENS in recipient mouse bowel. Importantly, human‐derived neurons form structural and functional connections with the endogenous ENS and thus are potentially capable of restoring function to neuropathic bowel. The data provide proof of concept for the in vivo transplantation of human ENCCs.

## Funding

NT is supported by the Great Ormond Street Hospital Children's Charity (GOSHCC). JC, DN, CM, JMD, and HG are funded through a GOSHCC grant awarded to NT. JC was part funded by grant from the Medical Research Council (G0800973) awarded to NT and AJB. This study was supported by the National Institute for Health Research Biomedical Research Centre at Great Ormond Street Hospital for Sick Children and University College London Institute of Child Health.

## Conflicts of Interest

The authors have no competing interests.

## Author Contribution

NT conceived and together with JC, DN, CM, and AJB designed the work; JC, DN, and CM together with SC and HG made substantial contributions to the acquisition, analysis, or interpretation of data for the work; JC, DN, CM, and NT helped draft the manuscript; NT and AJB revised it critically for important intellectual content. All authors contributed to the final approval of the version to be published and agreed to be accountable for all aspects of the work in ensuring that questions related to the accuracy or integrity of any part of the work are appropriately investigated and resolved.
